# *Pauca verba* on the association between protein intake and sarcopenia in older adults

**DOI:** 10.1016/j.jnha.2024.100448

**Published:** 2024-12-07

**Authors:** Hélio José Coelho-Júnior, Alejandro Álvarez-Bustos, Leocadio Rodriguez-Mañas, Francesco Landi, Emanuele Marzetti

**Affiliations:** a Fondazione Policlinico Universitario Agostino Gemelli, IRCCS, Rome, Italy; b Department of Geriatrics and Orthopedics, Università Cattolica del Sacro Cuore, Rome, Italy; c Biomedical Research Center Network for Frailty and Healthy Ageing (CIBERFES), Institute of Health Carlos III, Madrid, Spain; d Department of Geriatrics, Hospital Universitario de Getafe, Madrid, Spain; e Instituto de Investigación IdiPaz, Madrid, Spain

**Keywords:** Nutrition, Malnutrition, Physical function, Disability, Frailty

## Abstract

Sarcopenia is a prevalent neuromuscular condition among older adults, marked by significant reductions in muscle mass and strength, which result in notable impairments in physical performance. Modifications in lifestyle habits have been frequently highlighted as essential approaches to mitigate the progression of sarcopenia, with a particular focus on protein consumption. Over the past few decades, a wealth of knowledge has emerged, driven by both observational and experimental studies exploring various factors related to protein intake, such as amount, timing, and sources. This review provides a *pauca verba* overview of these findings, presenting a concise yet informative summary of key insights.

## 1 Presentation

**Fig. 1:**
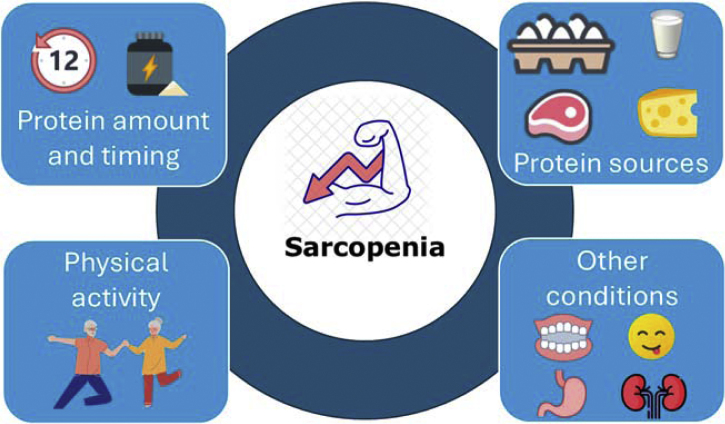
Protein intake associated-parameters and sarcopenia.

Sarcopenia, a prevalent muscular disease in older adults, is characterized by significant declines in muscle strength and mass, leading to severe impairments in physical performance [1,2]. Lifestyle modifications, particularly changes in dietary habits, have been widely proposed to counteract sarcopenia's progression, with a notable focus on protein intake [3]. Over recent decades, substantial research has examined various aspects of protein consumption, including quantity, distribution, and sources (Fig. 1). This mini-review provides a concise overview of current knowledge in this area.

## 2 Why do older adults need to consume more proteins?

Muscle protein synthesis (MPS) is stimulated by essential amino acids (AA), particularly branched-chain amino acids (BCAAs), obtained through adequate protein intake [4]. In younger muscles, BCAAs activate the mammalian target of rapamycin (mTOR) pathway and its downstream proteins, promoting muscle power synthesis (MPS) and supporting muscle anabolism [4]. However, as muscles age, higher levels of AA are needed to effectively stimulate MPS due to anabolic resistance, a condition where hyperaminoacidemia fails to fully promote MPS [4]. Additionally, while increased protein intake can enhance MPS in older adults, their maximum MPS capacity is reduced compared to younger individuals. This imbalance between MPS and muscle protein breakdown leads to the net loss of muscle mass [4].

These findings have prompted the development of specific protein intake guidelines for older adults to ensure an adequate supply of AA [3,4]. Current recommendations suggest a minimum daily protein intake of 0.8 g/kg of body weight (BW) for adults, including older individuals. However, these guidelines are based on nitrogen balance techniques, which have been criticized for their limitations in accurately assessing protein homeostasis [3,4]. Empirical evidence further challenges these recommendations, indicating that a minimum protein intake of 1.0 g/kg of BW/day is necessary to meet body protein requirements in a steady state [3,4].

As a result, older adults with insufficient protein intake may be at increased risk of developing muscle atrophy and, consequently, sarcopenia. Conversely, consuming dietary protein at levels exceeding current recommendations may help prevent or mitigate the progression of sarcopenia [3,4].

## 3 Do older adults with sarcopenia have a lower intake of proteins?

The theoretical framework suggests that low dietary protein intake may be linked to sarcopenia. While randomized clinical trials (RCTs) offer high-quality evidence on the impact of protein intake on sarcopenia, they often capture short-term, intervention-specific effects rather than habitual, long-term dietary patterns. In this context, observational studies play a crucial role in providing valuable insights into the relationship between protein consumption and sarcopenia.

A pooled analysis of over 3,000 community-dwelling older adults, with an average age of approximately 70 years, revealed that non-sarcopenic individuals had higher protein intake compared to their sarcopenic counterparts [5]. However, the analysis included only seven studies, limiting its generalizability. These findings are supported by meta-analyses examining the cross-sectional relationship between dietary protein intake and sarcopenia parameters [6,7]. Notably, older adults consuming ≥1.0 g/kg of BW/day demonstrated superior muscle strength, balance, mobility, and performance on the short physical performance battery (SPPB) compared to those with a protein intake below 0.8 g/kg of BW/day.

Longitudinal studies on protein intake and sarcopenia are limited. One notable investigation by Granic et al. [8] analyzed data from the Newcastle 85+ Study and found a positive association between high protein intake and the incidence of sarcopenia over three years, independent of physical activity levels. However, these results should be interpreted cautiously, as participants predominantly adhered to a traditional British diet rich in butter, red meat, gravy, and potatoes.

Although cross-sectional analyses provide promising insights, the scarcity of longitudinal studies investigations still hampers major conclusions

## 4 Are high protein diets effective in improving sarcopenia status in older adults?

Several RCTs have explored the effects of increased protein intake on sarcopenia parameters in older adults. A systematic review by Nunes et al. [9] analyzed studies involving protein supplementation or dietary protein augmentation in adults aged 18 and older. The findings revealed that increased protein intake alone did not significantly affect any sarcopenic domains.

In contrast, studies focusing exclusively on whey protein, a high-quality protein derived from cheese production, demonstrated important benefits. Protein supplementation significantly increased lean mass in individuals with sarcopenia [10,11], though no such improvements were observed in healthy adults [10]. Additionally, whey protein supplementation led to significant enhancements in physical performance [10,11]. The Intervention protocols varied widely, with oral doses ranging from 9.6 g to 40 g, administered once, twice, or three times daily [11].

The lack of significant effects in some studies may be attributed to the need for sufficient AA supply to be paired with resistance training to effectively stimulate muscle mass and neuromuscular function [9,11]. Additionally, many studies have been conducted on apparently healthy older adults, potentially limiting the applicability of findings to individuals with nutrient deficiencies or malnutrition, who might respond differently. It is also important to note that the effects observed in controlled interventions, particularly those involving supplements, may not easily translate to real-world clinical practice. Laboratory conditions and intervention protocols can be challenging to replicate outside a research setting, though such strategies might serve as an effective initial approach for patients with severe sarcopenia.

## 5 Protein intake and sarcopenia: beyond just quantity!

The association between protein intake and sarcopenia appears to depend not only on the quantity but also on the sources of protein (e.g., animal vs. plant-based) and its distribution throughout the day. Animal proteins generally have higher digestibility and contain greater concentrations of BCAAs, which are critical for optimizing MPS. Interestingly, Montiel-Rojas et al. [12] conducted one of the few studies exploring this relationship in older adults and found that substituting animal protein with plant-based protein was associated with a lower prevalence of sarcopenia. This suggests that the type of protein consumed may play a nuanced role in managing sarcopenia.

Protein distribution patterns have also been investigated in the context of sarcopenia, focusing on two primary approaches: *pulse-feeding*, where a large portion of protein is consumed in a single meal, and *spread feeding*, where protein intake is evenly distributed across meals. However, the evidence regarding the impact of these patterns on sarcopenia remains inconsistent, with no clear consensus on the optimal approach [6].

Addressing protein intake and its relationship with sarcopenia requires consideration of various factors beyond protein-specific parameters. These include the overall diet composition [13], the use of protein-enriched snacks [14], the presence of malnutrition [15], levels of physical activity [16], oral health [17], appetite [18], individual motivation [19], and cultural influences on dietary habits [20].

## 6 Conclusions

A strong biological rationale underpins the recommendation for higher protein intake to counteract sarcopenia in older adults. Intake levels exceeding current guidelines (≥0.8 g/kg of BW/day) appear essential for preserving or enhancing muscle mass and function, especially in those with sarcopenia and frailty. However, there is no substantial body of high-quality evidence to determine the optimal strategies for achieving these protein targets. Further research is urgently needed to investigate the impact of protein sources, distribution patterns, and other influencing factors, providing clearer guidance on addressing this important health challenge.

## CRediT authorship contribution statement

All authors contributed to the conceptualization, methodology, and writing of the original draft. HJCJ led the writing of the original draft and performed the review and editing of the manuscript. AAB and EM assisted in the review and editing of the manuscript. All authors contributed to the investigation, data analysis, and final approval of the manuscript.

## Declaration of generative AI in scientific writing

No Generative AI or AI -assisted technologies were used in the writing process.

## Funding

This work was supported by Innovative Medicine Initiative-Joint Undertaking (IMI-JU #115621).

## Data availability statement

Data used in the present manuscript are available in the cited articles. No new data were created for this study.

## Declaration of competing interest

The authors declare that they have no known competing financial interests or personal relationships that could have appeared to influence the work reported in this paper.
